# Intracellular Water Lifetime as a Tumor Biomarker to Monitor Doxorubicin Treatment *via* FFC-Relaxometry in a Breast Cancer Model

**DOI:** 10.3389/fonc.2021.778823

**Published:** 2021-12-03

**Authors:** Maria Rosaria Ruggiero, Simona Baroni, Valeria Bitonto, Roberto Ruiu, Smeralda Rapisarda, Silvio Aime, Simonetta Geninatti Crich

**Affiliations:** ^1^ Department of Molecular Biotechnology and Health Sciences, University of Turin, Turin, Italy; ^2^ IRCCS SDN, Naples, Italy

**Keywords:** NMR relaxometry, theranostics, doxorubicin, magnetic resonance imaging, cellular water efflux rate constant

## Abstract

This study aims to explore whether the water exchange rate constants in tumor cells can act as a hallmark of pathology status and a reporter of therapeutic outcomes. It has been shown, using 4T1 cell cultures and murine allografts, that an early assessment of the therapeutic effect of doxorubicin can be detected through changes in the cellular water efflux rate constant k_io._ The latter has been estimated by analyzing the magnetization recovery curve in standard NMR T_1_ measurements when there is a marked difference in the proton relaxation rate constants (R_1_) between the intra- and the extra-cellular compartments. In cellular studies, T_1_ measurements were carried out on a relaxometer working at 0.5 T, and the required difference in R_1_ between the two compartments was achieved *via* the addition of a paramagnetic agent into the extracellular compartment. For *in-vivo* experiments, the large difference in the R_1_ values of the two-compartments was achieved when the T_1_ measurements were carried out at low magnetic field strengths. This task was accomplished using a Fast Field Cycling (FFC) relaxometer that was properly modified to host a mouse in its probe head. The decrease in k_io_ upon the administration of doxorubicin is the result of the decreased activity of Na^+^/K^+^-ATPase, as shown in an independent test on the cellular uptake of Rb ions. The results reported herein suggest that k_io_ can be considered a non-invasive, early and predictive biomarker for the identification of responsive patients immediately from the first doxorubicin treatment.

## Introduction

Diagnostic imaging tools play a key role in the characterization of complex, heterogeneous and multifactorial diseases such as cancer. They are often crucial for the selection of the most suitable therapy and for the evaluation of its outcome, i.e. to increase the chance of success and reduce the side effects (1). The currently available pharmacological options in the field of breast cancer, which is the most common cancer in women, are under intense scrutiny to find means to obtain an early evaluation as to their efficacy. The World Health Organization (WHO) criteria and Response Evaluation Criteria in Solid Tumors (RECIST) are based on the assessment of tumor size in morphological images by Computer Tomography (CT) and Magnetic Resonance Imaging (MRI) (2, 3). However, volume changes are quite late events and their use in the evaluation of the undertaken therapy may be an issue when dealing with a disease for which time is a very important parameter. Therefore, there is a continuous search for methods that can report the early effects of therapy, in particular those that use biomarkers to report changes in tumor metabolic activity. Currently, metabolic assessments that are based on the use of positron emission tomography (PET) tracers are widely used as they reflect the viability of cancer cells and other functional changes in the response to anticancer treatments (4, 5). Moreover, the availability of advanced functional magnetic resonance imaging (fMRI) techniques has furnished new possibilities in the monitoring of therapeutic responses (6). There is a growing consensus that MRI protocols may provide powerful diagnostic tools in the field of oncology (7). Their diagnostic power arises primarily from disease-induced changes in the nuclear magnetic resonance (NMR) relaxation times of tissue, especially in the spin-lattice relaxation time T_1_ (8, 9). However, these changes cannot be fully exploited at the magnetic field strengths of currently available MRI scanners, as changes in tissue T_1_ do not appear to be sensitive enough at these field strengths to report on the response of the tumor cells to therapy. On the other hand, our group has recently demonstrated that measuring T_1_ at lower magnetic field strengths can be very informative and report on the tumor metabolic status (10, 11). This is possible because tissue T_1_ varies with applied magnetic field strength, with the differences between tissues being higher at lower magnetic field strengths (12–15). This behavior (often referred to as “T_1_-dispersion”, or simply relaxometry) is a marker of disease, but is invisible to conventional, fixed-field MRI scanners. Fast Field-Cycling (FFC)-NMR is the only practicable technique for measuring T_1_-dispersion. It involves switching the magnetic field between different field strengths (from 0.2 to 200 mT, typically) during the measurement procedure (16). The proton relaxation rate constants (R_1_ = 1/T_1_) of tissues are dependent on the extra/intracellular water ratio and the water exchange rate between the two compartments (10). Both terms are affected by tumor-cell metabolism. The relative amount of water in the two compartments and their exchange across cellular membranes are significantly different in healthy and tumor tissues. Recent studies have shown that the osmosis- and metabolism-driven movement of free water molecules across membranes, which affect cell volume and shape, may be an intrinsic and extremely sensitive reporter of pathology and its energetic deregulation (17–20).

In our previous work (10, 11), we demonstrated that observing R_1_ at low magnetic fields can clearly discriminate between healthy and tumor tissues as well as between different types of adenocarcinoma breast cancer. The differences in the observed relaxometric behavior can be accounted for in terms of the cellular water efflux rate constant, k_io_, that can be obtained from fitting the Magnetization decay acquired on the FFC-NMR relaxometer. This observation reports on the particular characteristics of the given tumor cell type. A smaller k_io_ indicates a slower water exchange rate across the transcytolemmal membrane, and this has been shown to be related to the overexpression/upregulation of the GLUT1 and Na^+^/K^+^-ATPase transporters.

On this basis, FFC-NMR is expected to provide relevant information about the metabolic mechanisms involved in disease progression and response to treatment. We hypothesize that k_io_ may be used as an early predictive biomarker of response to treatment. In this study, we have performed *in-vitro* and *in-vivo* experiments to evaluate how changes in k_io_ are related to treatment with doxorubicin, an anthracyline drug, that is routinely used in the treatment of several cancers including breast, lung, gastric, ovarian, thyroid, non-Hodgkin’s and Hodgkin’s lymphoma, multiple myeloma, sarcoma and pediatric cancers (21).

## Material and Methods

### Cell Cultures

The 4T1 (ATCC^®^ CRL-2539™) cell line, initially derived from spontaneous breast tumor growth in a BALB/c, was purchased from American Type Culture Collection (ATCC, USA). 4T1-R was derived from 4T1 (ATCC^®^ CRL-2539™). They were grown in RPMI 1640 medium supplemented with 10% fetal bovine serum (FBS), 100 U/mL Penicillin with 100 μg/ml Streptomycin, and 4 mM glutamine. Cells were cultured in 5% CO_2_/95% air at 37°C in a humidified chamber and were split every 2 to 3 days. All cells tested negative for mycoplasma using the MycoAlert™ Mycoplasma Detection Kit. All materials were purchased from Lonza (Basel, Switzerland).

### Drug-Resistant Cell Line (4T1-R)

4T1-R was derived from 4T1 (ATCC^®^ CRL-2539™), which was treated for six months with an increased concentration of doxorubicin (Sigma Aldrich) (from 50 nM to 5 μM) (22–24). At the confluence of 80%, the cells were seeded for the next treatment cycle with a higher concentration of the drug. The acquisition of drug resistance was evaluated using an MTT assay, based on the reduction of tetrazolium salts to formazan by mitochondrial succinate dehydrogenase, and quantified spectrophotometrically. The cells were seeded in 96 multi-well plates at a density of 10000 cells/well. After 24 h, doxorubicin (Sigma Aldrich) (1 to 50 μM)) was added to the cell suspensions, which were then incubated for a further 24 h at 37°C and 5% CO_2_. The medium was then removed and 100 μl of Thiazolyl Blue Tetrazolium Bromide (Sigma Aldrich) was added at a concentration of 0.45 mg/ml into each well and the plate was incubated for 4 h at 37°C and 5% CO_2_. After the removal of the suspension medium, 150 μl of dimethyl sulfoxide (DMSO) was added to each well to solubilize the formazan salt crystals that were produced by the metabolism of the live cells. After 30 minutes, the absorbance was read at 570 nm using an iMark microplate reader (Biorad). Cell vitality was reported as the percentage of dead cells relative to the control. The experiment was performed in quadruplicate and the data were graphically presented as mean ± SD.

### Western Blot

Frozen cells, that had not previously been treated with trypsin for detachment, were incubated in RIPA lysis buffer (150 mM NaCl; 50 mM Tris-HCl, pH 8.0; sodium dodecyl sulphate (SDS) 0,1%; sodium deoxycholate 0,5%; Nonidet P-40 1%) supplemented with 1 mM PMSF, 1 mM NaVO_4_, 1 mM NaF and protease-inhibitor cocktail (Sigma-Aldrich) for 40 min on ice. Cell lysates were centrifuged for 10 min at 14.000 g and the supernatant was harvested for quantification. The total protein concentration was quantified using the Pierce™ BCA Protein Assay Kit (Thermo-Fisher Scientific). After 30 min incubation at room temperature (when not otherwise specified) in β-mercaptoethanol-containing Laemmli Sample Buffer (Bio-Rad), equal amounts of protein lysates (ranging between 30 and 70 μg) were separated *via* electrophoresis in a 4-15% Mini-Protean TGX precast gel (Bio-Rad) and then transferred onto an Immobilion-P PVDF membrane (0.45 μm pore size, Merck Millipore). After blocking with either 5% non-fat dry milk (Santa Cruz Biotechnology) or 5% BSA (Sigma-Aldrich) in wash buffer (Tris Buffered Saline supplemented with 0.1% Tween-20 - T-TBS - from Sigma-Aldrich), the membranes were incubated overnight at 4°C with either rabbit anti-MDR1 (Cat#sc-9313, clone H-241 Santa Cruz Biotechnology, 1:250 in PBS, 1% BSA) or mouse anti-β-actin (Cat#sc-69879, Clone AC-15, Santa Cruz Biotechnology, 1:200 in T-TBS, 5% Milk) antibodies in blocking buffer. The membranes were then rinsed 3 times in T-TBS and incubated for 1 hour at room temperature with either HRP-conjugated anti-rabbit (Cat#A0545, Sigma-Aldrich, 1:2000 in T-TBS, 5% Milk) or anti-mouse (Cat#A4416, Sigma-Aldrich, 1:2000 in T-TBS, 5% Milk). β-actin was used as the loading control. The membranes were incubated with Pierce^®^ ECL Western Blotting Substrate (Thermo-Fisher Scientific) and images were acquired using a ChemiDoc™ Touch Imaging System (Bio-Rad).

### *In-Vitro* Determination of Cellular Membrane Water Exchange Rate Constants by a Relaxometric Procedure After Doxorubicin Treatment

The determination of cellular membrane water exchange rate constants was performed following the protocol described by Ruggiero et al. (10, 11). 4T1 and 4T1-R were seeded in a 175 cm^2^ flask at a density of 6 million cells/flask. After 24 h, cells were incubated with 0, 0.1, 0.5, 1, 5 µM of doxorubicin for a further 24 h (25, 26). The cells were detached using 0.05% trypsin and 0.02% EDTA, washed once with Phosphate Buffer Saline (PBS) and re-suspended in the presence of 10 mM Gd-HPDO3A (Prohance, kindly provided by Bracco S.p.A. (Milan, Italy)) in PBS. The relaxometric measurements were carried out within 15 min, during which cells were transferred to 5 mm NMR tubes and centrifuged for 5 minutes at 0.1 rcf (4°C). The water proton T_1_ values of the cellular pellets were measured at 0.5 T and 25°C on a Stelar SPINMASTER spectrometer (Stelar, Mede, Italy) using the inversion-recovery (IR) pulse sequence with 64 τ increments. The apparently biexponential recovery of the magnetization was observed. The water exchange rate constants (efflux, k_io_ and influx, k_oi_) across the cell membrane were determined by analyzing the IR data using the 2SX model (17, 18).

### Determination of Rb Uptake

Seven thousand and fifty cells were plated in 10 cm Ø dishes. After 24 h, they are incubated with different concentrations of doxorubicin (from 0 to 5 µM) for a further 24 h. They were then incubated for 1 h with 0.12 mM RbCl in Earle’s Balanced Salt Solution (EBSS) at 37°C and 5% CO_2_. Following four washing steps with cold PBS to remove the extracellular RbCl, the cells were harvested. A Bradford assay was used to determine the protein concentration before acidic digestion. 200 μl of the cell suspension and 200 μl of HNO_3_ (70%) were subjected to acidic mineralization to oxidize the organic matter, solubilize all metals and simplify the matrix. The microwave (ETHOS UP Milestone, Bergamo, Italy) heating program consisted of a ramp to 150°C in 6 minutes, followed by 8 min to 150°C. After mineralization, sample volumes were brought to 3 ml with doubly deionized water. The quantification of Rb was performed *via* ICP-MS (Element-2; Thermo-Finnigan, Rodano (MI), Italy) analysis. The calibration curve was obtained using absorption standard solutions (Sigma-Aldrich) in the range 0.2–0.005 μg/mL.

### Flow Cytometry

Having performed enzymatic detachment and several washings, the cells were resuspended in Annexin V binding buffer (Annexin V Apoptosis Detection Kit APC, eBioscience™) and incubated in flow cytometry tubes with the Annexin V-APC reagent, according to manufacturer’s instructions. After 15 min incubation at room temperature, the cells were rinsed in Annexin V binding buffer, the tubes were centrifuged and the supernatant was discarded. DAPI (1 µg/mL; Sigma Aldrich) was added to the tubes before acquisition with a flow cytometer. The fluorescence intensity and distribution of the cells were measured using a BD FACSVerse™ flow cytometer (BD - *Becton*, *Dickinson*, and Company) and the data were analyzed using FlowJo (LLC) software. Doxorubicin incorporation was estimated based on the mean fluorescence intensity (MFI) that resulted from doxorubicin intrinsic fluorescence (λ_Ex_ 470 nm, λ_Em_ 585 nm), which can be excited with a blue laser (488 nm) and detected using a PE (586/42 nm) filter. Cells that displayed negative staining for both Annexin V-APC and DAPI were considered to be live cells, those that were staining positive for Annexin V-APC, but negative for DAPI were considered early apoptotic. Those that were staining double positive were considered late apoptotic, whereas those staining positive for DAPI only were considered dead, but not apoptotic. Appropriate negative and positive controls were used.

### Animal Models

8-week-old female BALB/c mice were inoculated in their hind-limb muscle with 1 million 4T1 cells suspended in 100 μl of Phosphate Buffered Saline (PBS). BALB/c mice (Charles River Laboratories Italia S.r.l., Calco Italia) were maintained under specific pathogen-free conditions in the animal facility at the Molecular Biotechnology Center, University of Turin, and treated in accordance with EU (EU2010/63) and Italian (d.lgs 26/2014) regulations. Before undergoing imaging and nuclear magnetic resonance experiments, the mice were anesthetized with a mixture of tiletamine/zolazepam (Zoletil 100; Vibac, Milan, Italy) 20 mg/kg and xylazine (Rompun; Bayer, Milan, Italy) 5 mg/kg. The animal treatment protocol was approved by the Italian Ministry of Health (authorization number 807/2017-PR). When the tumor reached volumes of around 150-200 mm^3^, the animals were treated three times at 1-day intervals (days 0, 2 and 4) intravenously *via* the tail vein at a dose of 5 mg/kg of doxorubicin (26–28).

### Magnetic Resonance Imaging

T_2_-weighted images were acquired at 1 T on an Aspect M2-High Performance MRI System (Aspect Magnet Technologies Ltd., Netanya, Israel) consisting of a NdFeB magnet, equipped with a 35 mm solenoid Tx/Tr coil with an inner diameter of 35 mm. This system is equipped with fast gradient coils (gradient strength, 450 mT m^-1^ at 60 A; ramp time, 250 μs at 160 V) with a field homogeneity of 0.2−0.5 G. MR images were acquired using a Fast Spin Echo sequence (FSE) (TR 3000 ms; TE 50 ms; number of slices 11; slice thickness 1 mm; FOV 50x50 mm; matrix 168 × 160, Flip angle 90°). Tumor volume was determined using ITK-SNAP software.

### *In-Vivo* Nuclear Magnetic Resonance Dispersion Profiles (NMRD)

*In-vivo* NMRD profiles were acquired on the Stelar FFC-NMR relaxometer (Stelar S.n.c., Mede (PV), Italy), which is equipped with a 40 mm 0.5 T FC magnet and a dedicated 11 mm solenoid detection coil. The relaxometer operates under complete computer control with an absolute uncertainty in the 1/T_1_ value of ±2%. The typical field sequences used were the Non-Polarized sequence (NP/S), between 16 and 7 MHz, and the Pre-Polarized sequence (PP/S), between 7 and 0.01 MHz. The observation field was set at 14.5 MHz, while the polarization field was set at 13 MHz. Acquisition was performed using 32 τ over a long-time range (2.8-4 s) in order to sample both the rapidly and slowly relaxing magnetization components. These profiles were acquired at seven different relaxation field strengths (0.01, 0.02, 0.037, 0.07, 0.15, 0.39, 1 MHz in terms of proton Larmor frequency). During acquisition, murine temperature was maintained at 25°C using a gel pad. Data were simultaneously analyzed using Origin software (OriginPro 8.5.0 SR1, OriginLab, Northampton, MA, Levenberg-Marquardt algorithm, RRID : SCR_014212), in accordance with the 2SX model (10, 17, 18), sharing the extracellular volume fraction, V_ex_, and the extracellular water life time constant, τ_ex_, parameters and maintaining the extracellular relaxation rate constant, R_1ex_, fixed to the Matrigel values obtained in a separated experiment (10). The V_ex_ value was allowed to vary within a reliable range as reported in the literature; 0.09-0.19 for healthy mouse hind limbs, and 0.15-0.5 for tumor mouse hind limbs (18–20, 29, 30).

### Tunel Assay

Immediately after the NMRD profile was acquired (3 days after the first doxorubicin treatment), the mice were sacrificed and perfused, *via* the vascular system, with a 4% paraformaldehyde solution as a fixation procedure to obtain the best possible preservation of the tumor tissue for immunohistochemistry (31). PFA-fixed and paraffin-embedded Dewaxed 5 μm tumor sections were stained with the *in-situ* Apoptosis Detection Kit (Abcam), according to manufacturer’s protocol.

## Results and Discussion

### *In-Vitro* Experiments on Murine Adenocarcinoma Cells (4T1)

Murine adenocarcinoma 4T1 cells were treated with doxorubicin (0.1-5 µM) for 24 h. The quantitative determination of the cellular water efflux rate constant (k_io_) was carried out by following a well-established relaxometry-based method at 0.5 T in the presence of a 10 mM solution of the paramagnetic Gd-HPDO3A complex in the extracellular space of the cellular suspension (10, 32–34). The extracellular distribution of the paramagnetic agent allows a marked difference in proton relaxation rates to be achieved between the extra- and the intra-cellular compartments. It follows that the recovery of the magnetization curve after its selective inversion is characterized by an apparently bi-exponential behavior, whose fitting yields estimated values for k_io._

**Figure 1:**
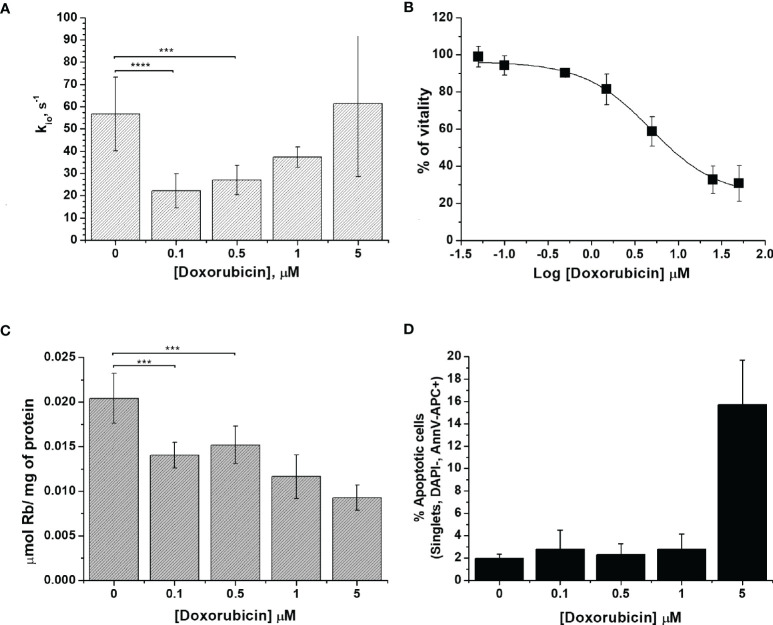
The characterization of 4T1 cells. **(A)** The cellular water efflux rate constant, k_io_ (s^-1^), as determined 24 h after doxorubicin treatment at different drug concentrations (0.1-5 µM). k_io_ was determined by measuring the water proton relaxation times of cell suspensions in the presence of 10 mM of Gd-HPDO3A at 25°C on a fixed frequency spectrometer operating at 0.5 T. Data are expressed as the mean ± SD with n ≥7. **(B)** Percentage of viable cells plotted vs. Log[Doxorubicin] after 24 h of treatment, obtained using the MTT test. Data are expressed as the mean ± SD of three independent experiments. **(C)** Cellular uptake of Rb (µmol of Rb/mg of protein) measured 24 h after doxorubicin treatment. The cells were suspended in a medium containing RbCl 0.12 mM for 1 h at 37°C and 5% of CO_2_. Data are expressed as the mean ± SD of three independent experiments. **(D)** Determination of the % of apoptotic cells after doxorubicin treatment at different concentrations. The percentage of apoptotic cells was assessed using DAPI-, AnnV-APC+ staining with FACS analysis. Data are expressed as the mean ± SD of three independent experiments. (***p ≤ 0.001, ****p ≤ 0.0001).

In **Figure 1A**, the obtained k_io_ values are plotted as a function of the doxorubicin concentrations. The observed variations were statistically significant at low doses of the drug (0.1 and 0.5 μM) with P-values of 0.0000499 and 0.000215, respectively. Moreover, at the same doxorubicin concentrations, the MTT test showed that the treated cells had high vitality, which is very similar to the results found in the control experiment (**Figure 1B**). This finding is taken as a good indicator to support the view that k_io_ variations could be considered to be an early predictive biomarker of treatment response.

We surmise that the observed behavior, induced by the presence of increasing doses of the drug, can be accounted for in terms of the cellular system being subjected to a variety of different effects. In the experiments at low drug concentrations, the main biologically detectable phenomenon is a decrease in k_io_, which is, in principle, associated with an alteration of the membrane transport system, which is likely due to a decrease in Na^+^/K^+^-ATPase activity, as previously reported (10). In the case of the experiments at higher concentrations of the drug, one has to deal with extensive transformations that eventually lead to cell death. In cancer cells, doxorubicin acts *via* two principal mechanisms: i) intercalation into DNA and the disruption of topoisomerase-II-mediated DNA repair, with the consequent generation of free radicals, which damage lipids, DNA and proteins (21); and, ii) the inhibition of membrane-associated ion pumps, which is induced by the major metabolite of doxorubicin, doxorubicinol (21, 35–37). In principle, the two mechanisms can have opposite effects on k_io_. The transport activity of Na^+^/K^+^-ATPase was assessed to provide more insight into the doxorubicin response. The method (38), relies on the ICP-MS quantification of the cellular uptake of the rubidium ion, Rb^+^, which is an established congener for K^+^ transport by Na^+^/K^+^-ATPase (38, 39). It is well established that this ICP-MS method provides great accuracy in monitoring metal-ion transporters in a wide range of cell types and conditions (40). After doxorubicin treatment, adherent cells were incubated in the presence of RbCl 0.12 mM for 1 h at 37°C and 5% of CO_2_. The uptake of Rb, normalized to the amount of cell proteins, decreased significantly even in cells treated with the lowest doses of the drug (i.e. when the concentrations were 0.1 and 0.5 µM, respectively, with a P-value <0.01) (**Figure 1C**). The decrease in Rb uptake supports the view that the first effect induced in the treated cells is a reduced Na^+^/K^+^-ATPase efficiency, which, in turn, is responsible for the observed decrease in water exchange across the cellular membrane. For 4T1 cells, the amount of Rb per cell decreased by 30% and 54% upon changing from 0.1 to 5 µM doxorubicin treatment, respectively.

The response to doxorubicin in terms of cell deaths was assessed next. **Figure 1D** reports the percentages of apoptotic cells as a function of the applied doxorubicin concentrations. From these results, it is evident that apoptosis occurs only at higher drug concentrations, which corroborates our working hypothesis according to which k_io_ variations report early changes in cellular activity following doxorubicin treatment.

### *In Vitro* Experiments on Doxorubicin-Resistant 4T1 Cells (4T1-R)

**Figure 2:**
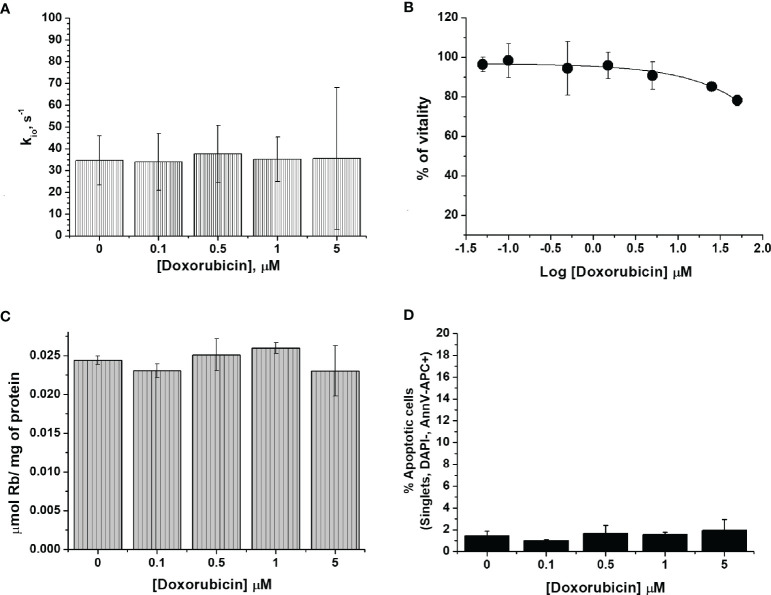
The characterization of 4T1-R cells. **(A)** The cellular water efflux rate constant, k_io_ (s^-1^), determined 24 h after doxorubicin treatment at different drug concentrations (0.1-5 µM). k_io_ values were determined by measuring the water proton relaxation times of cell suspensions in the presence of 10 mM of Gd-HPDO3A at 25°C on a fixed frequency spectrometer operating at 0.5 T. Data are expressed as the mean ± SD with n= 4. **(B)** Percentage of viable cells as determined using the MTT test and plotted vs Log[Doxorubicin] µM. Data are expressed as the mean ± SD of three independent experiments. **(C)** Cellular uptake of Rb (µmol of Rb/mg of protein) measured 24 h after doxorubicin treatment. The cells were suspended in a medium containing RbCl 0.12 mM for 1 h at 37°C and 5% of CO_2_. Data are expressed as the mean ± SD of three independent experiments. **(D)** Determination of the % of apoptotic cells after doxorubicin treatment at different concentrations. The percentage of apoptotic cells was assessed by DAPI-, AnnV-APC+ staining using FACS analysis. Data are expressed as the mean ± SD of three independent experiments.

One of the major drawbacks of chemotherapy is drug resistance. To mimic the acquisition of drug resistance (25), the murine mammary carcinoma 4T1 cells were treated with doxorubicin for several cycles with an increased concentration of doxorubicin (from 50 nM to 1 µM). After six months of treatment, the cells became resistant to doxorubicin as assessed by the results of the vitality test (i.e. MTT) (**Figure 2B**).

In a previous work, the acquisition of drug resistance was ascribed to the overexpression of ATP binding transporters, such as the MDR1/P-glycoprotein (Pgp, encoded by the *ABCB1* gene) (25, 41). We can confirm this notion as the western blot analysis of the MDR1/P-glycoprotein (**Figure S1**), using both cytoplasmic and nuclear extracts of 4T1 and 4T1-R cells, reported the overexpression of this glycoprotein in the resistant cell line.

The detection of doxorubicin fluorescence is a good reporter of the intracellular incorporation of the drug. **Figure S2** shows that the fluorescent response was concentration-dependent and significantly higher in 4T1 than in 4T1-R cells. Further support was gained by plotting the mean fluorescence intensity (MFI) as a function of doxorubicin concentration in the incubation medium. The results confirmed the increased doxorubicin uptake in wild type 4T1 cells compared to the resistant clone (**Figure S2**).

Subsequently, we investigated whether the administration of doxorubicin has an effect on the transport of water molecules across the membrane of 4T1-R cells (**Figure 2A**). The obtained k_io_ values showed a non-significant decrease and remained almost constant over the entire range of investigated doxorubicin concentrations. These results support the view that the k_io_ decrease in 4T1 cells observed at the low drug concentrations is due to the specific cytotoxic effect that is induced by doxorubicin, and not to an aspecific interaction between doxorubicin and the cell membrane that may potentially cause a change in cell-membrane permeability, which, most likely, would also have been present in the 4T1-R cells. The ICP-MS quantification of Rb confirmed that the transport activity of Na^+^/K^+^-ATPase in 4T1-R was not affected by the administration of doxorubicin. The uptake of Rb ions, normalized to the amount of cell proteins, was not significantly different in the cells treated with different concentrations (0.1 and 5 µM) of the drug, compared to control cells (**Figure 2C**). The determination of the percentage of apoptotic cells by FACS analysis confirmed that the 4T1-R resistant cell line was not affected by the toxicity of the drug. (**Figure 2D**).

### *In-Vivo* Experiments: Relaxometric Assessment of Doxorubicin Treatment in Balb/c Mice Bearing 4T1 Tumors

**Figure 3:**
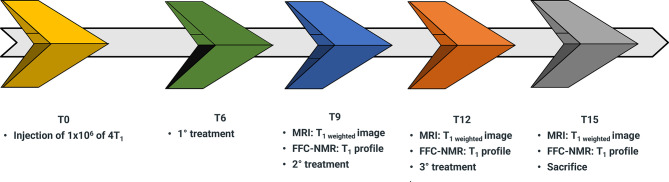
Schematic representation of the workflow for the assessment of doxorubicin treatment in 4T1 tumor animal models over a time course of 15 days (T0-T15).

To verify the hypothesis that the assessment of k_io_ may be used as an early predictive biomarker of treatment response *in-vivo*, an experimental setup was designed for mammary-tumor-bearing mice (**Figure 3**). The determination of k_io_, *via* the acquisition of the FFC-NMR profiles of the tumor region, was carried out before, during and after drug treatment.

4T1 cells (1x10^6^) were injected into the hind-limb muscle to obtain the corresponding tumor xenograft for the *in-vivo* acquisition of proton relaxation rates at low and variable magnetic fields. This was carried out using a FFC-relaxometer that was equipped with a wide bore magnet (40 mm diameter) that can host a small mouse. The position of the graft was dictated by the circular shape of the detection coil and its 11 mm diameter (10, 42). Due to the absence of spatial discrimination, the acquisitions were only carried out on mice bearing tumors with a volume > 60% of the total leg. Starting from the sixth day (T6 in the workflow sketch of **Figure 3**) from injection, mice (n= 7) were treated with 5 mg/kg of doxorubicin or with the vehicle (control mice n=7). The treatment was repeated twice more at three-day intervals. The relaxation profiles were acquired in the range 0.01-10 MHz three days after each treatment.

**Figure 4:**
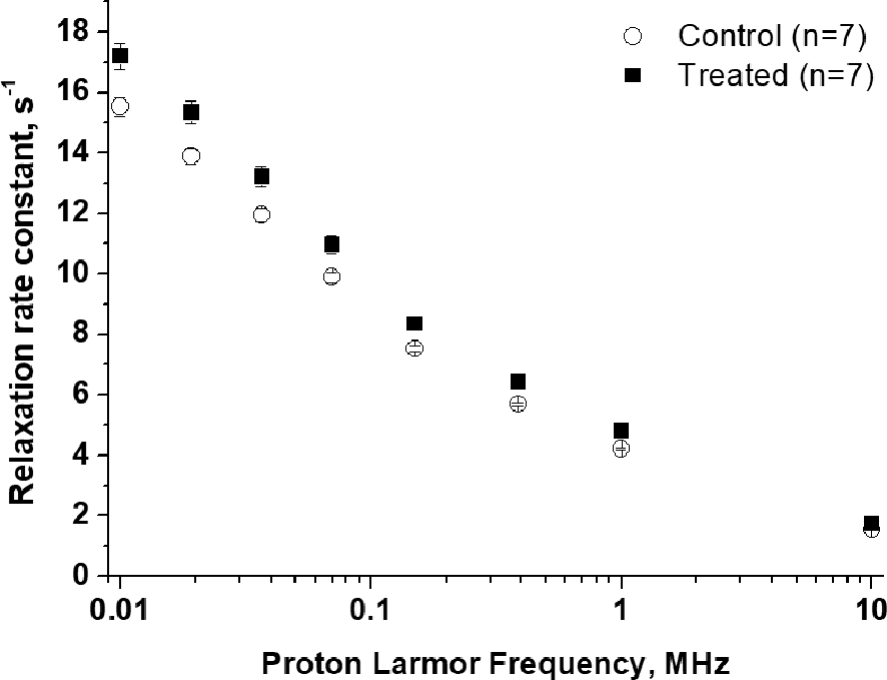
1/T_1_^1^H-NMRD profiles of tumor-bearing mouse legs: treated animals (n=7), black square, compared to control animals (n=7), empty circle. Error bars represent the SE of the experimental data. (p value at 0.01 MHz = 0.0078).

**Figure 5:**
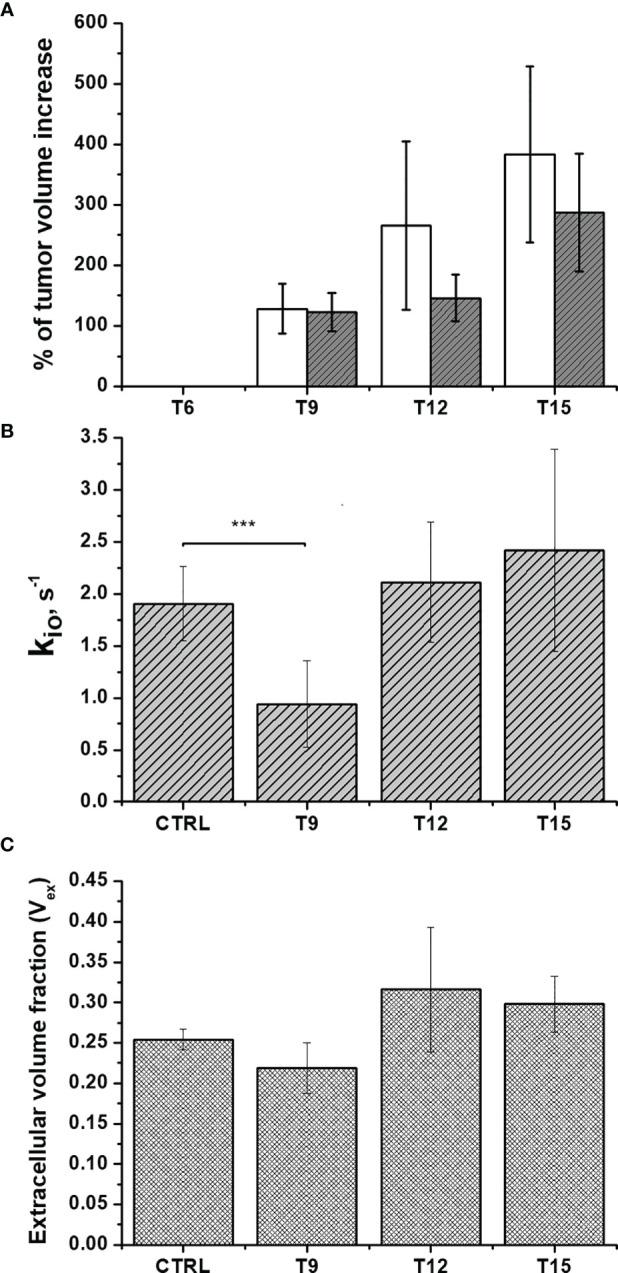
*In-vivo* assessment of doxorubicin treatment in Balb/c mice that bear 4T1 tumors: **(A)** The percentage of volume increase in the tumor region as determined by T_2_-weighted images acquired at 1 T using the ITK-SNAP software. Open and filled bars correspond to untreated and treated mice, respectively; **(B)** The cellular water efflux rate constant, k_io_ (s^-1^) and **(C)** The extra-cellular volume fractions (V_ex_) were calculated *via* the analysis of the multi-relaxation rate measurements in the range 0.01-1MHz using the 2SX model for treated animals (n=8) compared to control animals (n=7). Error bars represent the SD of the experimental data. (***p ≤ 0.001).

The acquisition of each 1/T_1_^1^H-NMRD profile took about 20 minutes and the temperature of the mice was maintained using a gel pad heated at 37°C. **Figure 4** shows a comparison of the relaxation profiles acquired in control (untreated tumor) and doxorubicin-treated mice. Even after the first treatment, a significant increase in the relaxation rate constants was observed for the treated mice (P-value at 0.01 MHz = 0.00779). The larger R_1_ values observed for the treated mice reflect the slower water exchange (corresponding to a smaller k_io_) between the intra- and extracellular compartments, as seen in the *in-vitro* experiments reported above. Here again, treatment with doxorubicin first resulted in a decrease of k_io_ (due to damage induced in the transporting system) that, in turn, leads to an increase in the measured R_1_ values. The smaller R_1_ value of the largest intra-cellular compartment dominates the observed R_1_ in the presence of limited exchange between the two compartments. Interestingly, the effect of doxorubicin treatment was not detectable in T_2_-weighted MR images acquired at 1 T, as the tumor volume was not significantly different in the treated animals and controls (P > 0.05) (**Figure 5A** and **Figure S3**). This observation indicates that morphological changes need longer time to be detected thus hampering an early assessment of the therapeutic outcome.

It follows that, as reported previously (10, 11, 32), the differences in relaxation rate constants, observed at low magnetic fields, are related to the occurrence of different water exchange regimes between the intra and extra-cellular compartments in the tumor tissue. During the time of an NMR experiment (or in image acquisition), water molecules explore both compartments and the observed R_1_ results from a mixing of their relaxation rate constants (R_1in_ and R_1ex_) weighted by their respective volume fractions (V_in_ and V_ex_). Unlike with high-field measurements, the *in-vivo* acquisition of a relaxation dispersion profile between 0.01-1 MHz allows us to work with differences in the intra- and extra-cellular relaxation rate constants (R_1in_ and R_1ex_, respectively) that are large enough to make the magnetization decay directly dependent on k_io_ (10, 32, 42). As demonstrated both in the cell suspensions and *in-vivo* experiments, k_io_ appears to be a good biomarker of tumor-cell status that can be exploited to non-invasively monitor the early effects of doxorubicin treatment. Therefore, k_io_ can be measured *in vivo* using multi-relaxation rate measurements in the range 0.01-1 MHz and the 2SX model [6,18], without the need to use paramagnetic contrast agents. **Figure 5B** shows that a significant decrease (P=0.000207) in k_io_ was observed 3 days after the first treatment, providing an early indication of the effect of doxorubicin on cell metabolism and vitality.

**Figure 6:**
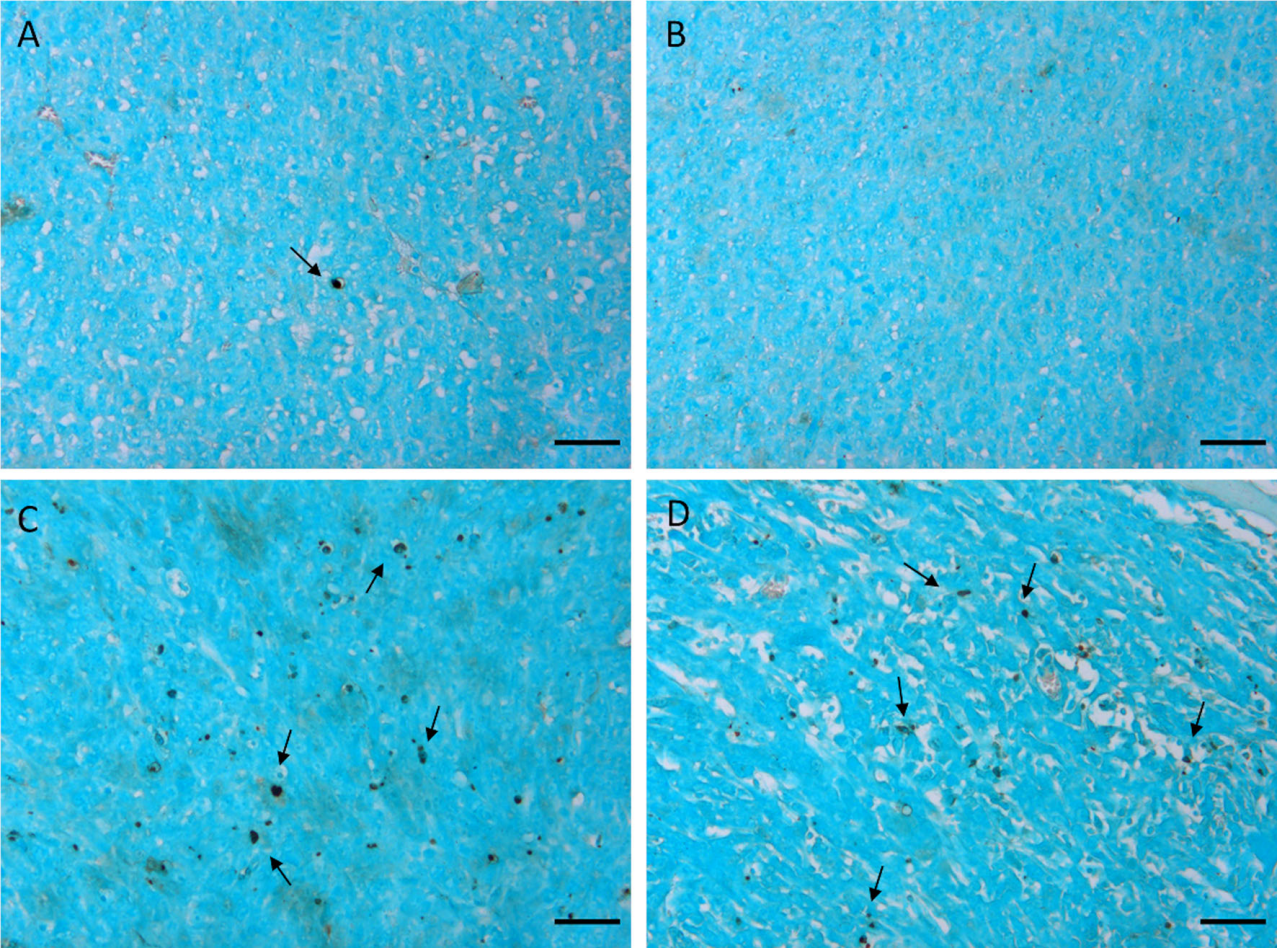
TUNEL staining of 4T1 tumors excised 3 days after the first treatment. Representative images of tumors treated with the vehicle (0.9% saline solution) **(A, B)** and with doxorubicin 5 mg/kg **(C, D)**. Arrows indicate apoptotic nuclei. Cells were counterstained with methyl green. Scale bars= 50 µm”.

As previously reported and herein confirmed in the Rb-uptake experiment, one of the important cytotoxic effects of this antitumor drug is its hampering of Na^+^/K^+^-ATPase activity. After the second and the third treatment, k_io_ returns to the initial value. We think that this result reflects the massive death of tumor cells, which completely permeabilizes the cytosolic membrane to water. Accordingly, **Figure 5C** shows an initial decrease in V_ex_, as a consequence of the decreased k_io_, followed by an increase in V_ex_ after the 2^nd^ and 3^rd^ treatments. The observed increase in V_ex_ is, again, a direct consequence of the cell death caused by doxorubicin. Once the membrane of the dead cells becomes fully permeable to water, the corresponding volume occupied by these cells becomes part of the V_ex_ compartment. These results are in good agreement with the histological apoptosis evaluation on the slices of tumors, that were recovered 3 days after the first treatment, using the TUNEL (terminal deoxynucleotidyl transferase-mediated deoxyuridine triphosphate nick‐end labeling) assay. **Figure 6** shows that there are more apoptotic cells in the treated sample than in the control, suggesting that both k_io_ and V_ex_ may act as biomarkers for the toxic effect of doxorubicin, significantly earlier than the macroscopic observation associated to the morphological changes related to slower tumor growth. Finally, it is important to notice that *in vivo* k_io_ magnitude is reduced by a factor of ~25, (**Figure 5B**) in respect to the *in vitro* values (**Figures 1** and **2**). There are many possible reasons to account for the observed behaviour. Cells within a tissue interact with neighbouring cells and with the extracellular matrix in a very different fashion in respect to what occurs in cellular pellets. This may result in significant modification of cell properties, size, morphology, elasticity, etc., i.e. properties that, in turn, may affect both passive and energetically driven components of water exchange across the cellular membrane. One may also expect that the high interstitial fluid pressure/flow and the low lymphatic drainage, in particular in the inner core of a solid tumor (43), may play a role in the translation from *in vivo* to *in vitro* studies. Likely the cells in culture take up more nutrients than in tissue and this would favour the view that the main determinant of the observed change relies on the increased metabolic activity of the tumour cells. Interestingly, upon comparing cell lines, characterized by different metabolic activities, it was found that the differences in kio observed *in vivo* in tumour tissues are maintained *in vitro* (10). Analogously, in this study, it was observed that the effect of doxorubicin treatment *in vivo* is maintained in the *in vitro* experiment

## Conclusion

In summary, the results reported herein show that relaxometric measurements using FFC-NMR on tumor specimens can provide an important diagnostic contribution for the early assessment of the therapeutic effects associated with the application of doxorubicin. This method is based on the exploitation of the changes in the MR proton water signal that are induced by water exchange rate across the cellular membrane, causing a variation in V_ex_. It appears to act as a hallmark that can report on the status of the tumor and its response to treatment. The herein-reported results suggest that k_io_ can be considered an early and predictive biomarker for the identification of responsive patients after the first treatment with doxorubicin. Even though FFC-NMR instrumentation is not endowed with spatial resolution, the knowledge obtained in this study can facilitate new diagnostic opportunities for the determination of therapeutic outcomes that would be possible with FFC MRI scanners. Two prototypes of human whole-body-sized FFC-MRI scanners have recently been built at Aberdeen University by Lurie and coworkers (44–46).

## Data Availability Statement

The original contributions presented in the study are included in the article/**Supplementary Material**. Further inquiries can be directed to the corresponding author.

## Ethics Statement

The animal treatment protocol was approved by the Italian Ministry of Health (authorization number 807/2017-PR).

## Author Contributions

Conceptualization, MR, SB, VB, SA, and SGC. Methodology, MR and SGC. Validation, MR, SB, VB, and RR. Formal analysis, MR, SB, VB, and SGC. Investigation, MR, SB, VB, RR, and SR. Resources, SGC. Writing—original draft preparation, MR, SB, VB, SA, and SGC. Writing—review and editing, MR, SB, SA, and SGC. Visualization, MR, VB, and SB. Supervision, SGC. Project administration, SGC. Funding acquisition, SA and SGC. All authors have read and agreed to the published version of the manuscript.

## Funding

This project has received funding from the European Union Horizon 2020 research and innovation program under grant agreement No 668119 (project IDentIFY). The Italian Ministry for Education and Research (MIUR) is gratefully acknowledged for yearly FOE funding to the EuroBioImaging Multi-Modal Molecular Imaging Italian Node (MMMI). Maria Rosaria Ruggiero was supported by a “FIRC-AIRC fellowship for Italy”.

## Conflict of Interest

The authors declare that the research was conducted in the absence of any commercial or financial relationships that could be construed as a potential conflict of interest.

## Publisher’s Note

All claims expressed in this article are solely those of the authors and do not necessarily represent those of their affiliated organizations, or those of the publisher, the editors and the reviewers. Any product that may be evaluated in this article, or claim that may be made by its manufacturer, is not guaranteed or endorsed by the publisher.
