# Supplementary material for: Intracellular Water Lifetime as a Tumor Biomarker to Monitor Doxorubicin Treatment via FFC-Relaxometry in a Breast Cancer Model
Source: Front Oncol. 2021 Dec 3;11:778823. doi: 10.3389/fonc.2021.778823 (PMC8678130; doi:10.3389/fonc.2021.778823)
Supplement: Supplementary file 1 [file DataSheet_1.docx]

**Supplementary materials**

**Intracellular Water Lifetime as a Tumour Biomarker to monitor Doxorubicin treatment *via* FFC-Relaxometry in a Breast Cancer Model**

Maria Rosaria Ruggiero^1^, Simona Baroni^1^, Valeria Bitonto^1^, Roberto Ruiu^1^, Smeralda Rapisarda^1^, Silvio Aime^2^, Simonetta Geninatti Crich^1^

^1^Department of Molecular Biotechnology and Health Sciences, University of Turin, Turin, Italy

^2^IRCCS SDN, Naples, Italy

**Western Blot**

Frozen cells, not previously treated with trypsin for detachment, were incubated in RIPA lysis buffer (150 mM NaCl; 50 mM Tris-HCl, pH 8.0; Sodium dodecyl sulphate (SDS) 0,1%; Sodium deoxycholate 0,5%; Nonidet P-40 1%) supplemented with 1 mM PMSF, 1 mM NaVO_4_, 1 mM NaF and protease inhibitors cocktail (Sigma-Aldrich) for 40 min on ice. Cell lysates were centrifuged for 10 min at 14.000 *g* and supernatant harvested for quantification. Total protein concentration was quantified using the Pierce™ BCA Protein Assay Kit (Thermo-Fisher Scientific). Following 30 min incubation at room temperature (when not otherwise specified) in β-mercaptoethanol-containing Laemmli Sample Buffer (Bio-Rad), equal amounts of protein lysates (ranging between 30 and 70 μg) were separated through electrophoresis in a 4-15% Mini-Protean TGX precast gel (Bio-Rad) and then transferred onto an Immobilion-P PVDF membrane (0.45 μm pore size, Merck Millipore). Following blocking with 5% non-fat dry milk (Santa Cruz Biotechnology) or 5% BSA (Sigma-Aldrich) in wash buffer (Tris Buffered Saline supplemented with 0.1% Tween-20 - T-TBS - from Sigma-Aldrich), membranes were incubated overnight at 4°C with rabbit anti-MDR1 (Cat#sc-9313, clone H-241 Santa Cruz Biotechnology, 1:250 in PBS, 1% BSA) or mouse anti-β-actin (Cat#sc-69879, Clone AC-15, Santa Cruz Biotechnology, 1:200 in T-TBS, 5% Milk) antibodies in blocking buffer. Membranes were then rinsed 3 times in T-TBS and then incubated for 1 hour at room temperature with HRP-conjugated anti-rabbit (Cat#A0545, Sigma-Aldrich, 1:2000 in T-TBS, 5% Milk) or anti-mouse (Cat#A4416, Sigma-Aldrich, 1:2000 in T-TBS, 5% Milk). β-actin was used as loading control. Membranes were incubated with Pierce® ECL Western Blotting Substrate (Thermo-Fisher Scientific) and images were acquired using a ChemiDoc^TM^ Touch Imaging System (Bio-Rad).


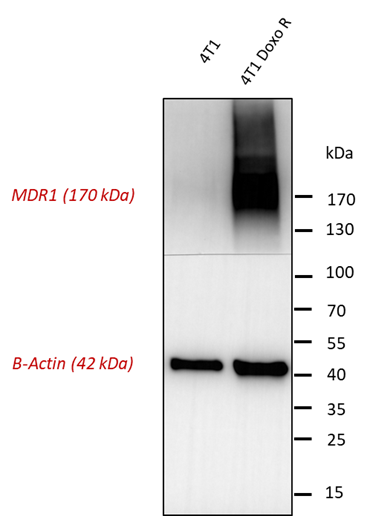


**Figure S1:** The western blot analysis of MDR1/P-glycoprotein, using both cytoplasmic and nuclear extracts of 4T1 and 4T1-R cells confirmed the overexpression in resistant cell lines.


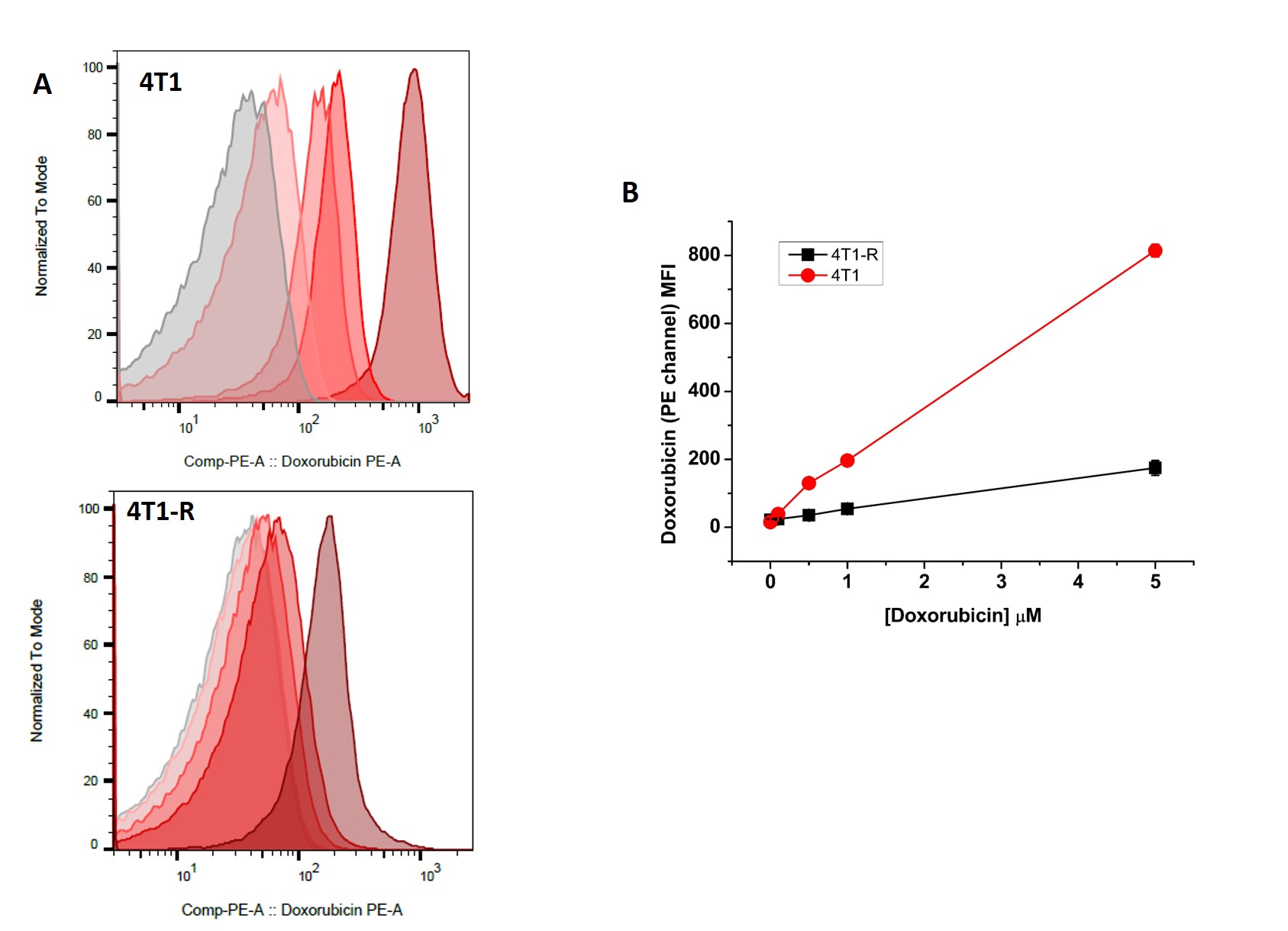


**Figure S2: A)** The phosphoethanolamine (PE) signal, due to doxorubicin fluorescence, is a good index of intracellular incorporation of doxorubicin showing that this signal changes as a function of drug concentration in 4T1-R than 4T1 only at high concentration of the drug. **B)** Further confirmation of their resistance comes from the graphs in which the mean fluorescence intensity (MFI) was plotted as a function of doxorubicin concentration. The results confirmed uptake increased of doxorubicin in wild type 4T1 than resistant ones.


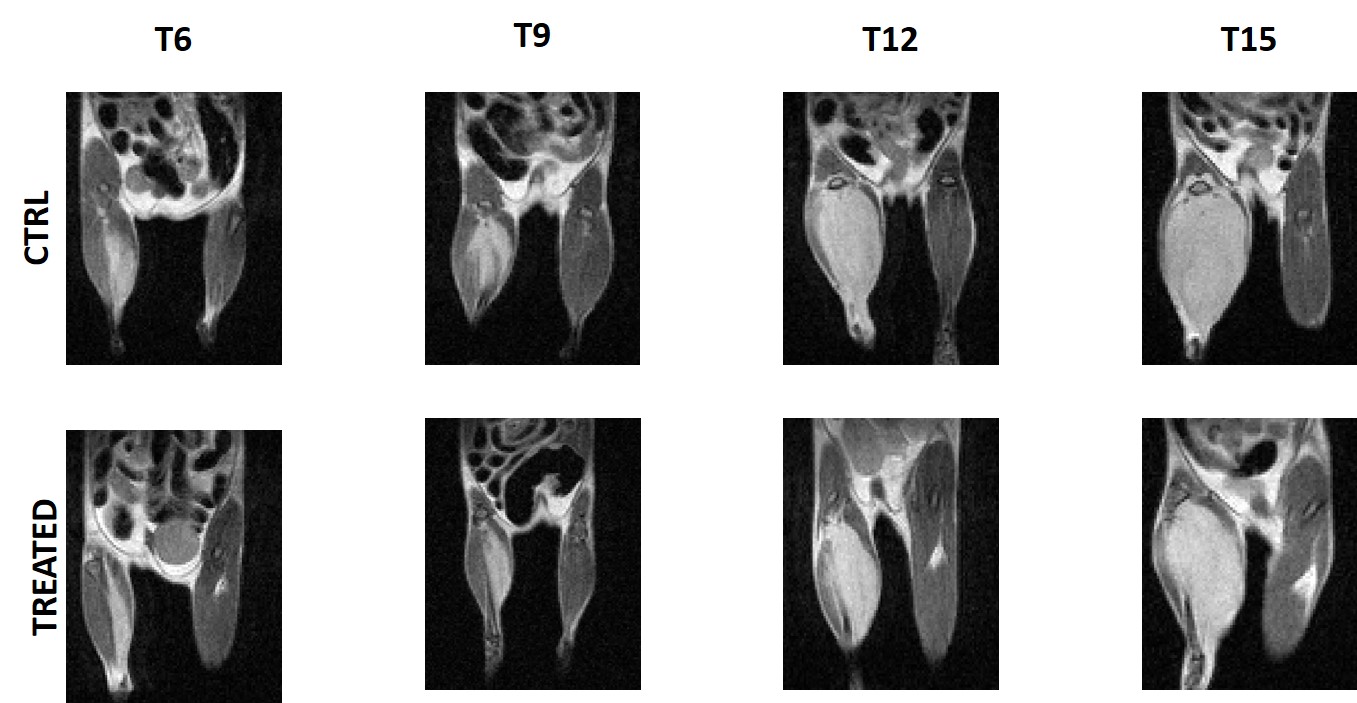


**Figure S3:** The T_1-weighted_ images were acquired at 1 T on Aspect M2-High Performance MRI System (Aspect Magnet Technologies Ltd., Netanya, Israel) every three days as described in figure 3 of the main text to assess the doxorubicin treatment (5 mg/kg) in 4T1 animal models compared to control animals (0.9% saline solution). Tumor volume was determined using the ITK-SNAP software and the results are shown in figure 5 A
